# Augmented Wound-Healing Effect of Sodium Thiosulfate-Infused Cosmetic Creams in Frostbite

**DOI:** 10.3390/pharmaceutics17121610

**Published:** 2025-12-15

**Authors:** George J. Dugbartey, Liam McFarlane, Tamara S. Ortas, Sally Major, Aaron Haig, Alp Sener

**Affiliations:** 1 Department of Surgery, Division of Urology, London Health Sciences Center, Western University, London, ON N6A 5A5, Canada; gdugbart@uwo.ca; 2 Matthew Mailing Center for Translational Transplant Studies, London Health Sciences Center, Western University, London, ON N6A 5A5, Canadasortas@uwo.ca (T.S.O.);; 3 Department of Physiology & Pharmacology, Western University, London, ON N6A 5C1, Canada; 4 Department of Physiology and Pharmacology, Accra College of Medicine, East Legon, Accra P.O. Box CT9828, Ghana; 5 Department of Microbiology & Immunology, Schulich School of Medicine & Dentistry, Western University, London, ON N6A 5A5, Canada; 6 Department of Pathology, Schulich School of Medicine & Dentistry, LHSC University Hospital, Western University, C4208, 339 Windermere Road, London, ON N6A 5A5, Canada

**Keywords:** frostbite injury, Aveeno, Dove, Neutrogena, Nivea, sodium thiosulfate, hydrogen sulfide (H_2_S)

## Abstract

**Background:** Frostbite injury is a thermal injury where ice crystals form in skin tissues and subsequently lead to damage due to prolonged exposure to cold temperatures below 0 °C. The extremities are mostly affected, leading to potential amputation. As there is no pharmacological treatment of frostbite injury, we recently reported that non-clinically viable hydrogen sulfide (H_2_S) donors promote frostbite wound healing in mice. In this study, we investigated whether commonly used cosmetic creams supplemented with sodium thiosulfate (STS), a clinically viable H_2_S donor drug, also promote healing of frostbite wounds. **Methods:** Frozen magnets (−80 °C) were placed on the dorsal skin of 40 C57BL/6 mice for 3 min to induce frostbite injury. Next, commercially available cosmetic creams (Aveeno, Dove, Neutrogena, and Nivea) were topically applied on frostbite wounds daily for 14 days with or without 150 µM of STS supplementation. The mice were sacrificed on day 15 after induction of frostbite injury, and samples of the injured dorsal skin tissue were collected for analysis. **Results:** Addition of STS enhanced frostbite wound healing, as evidenced by progressive and significantly reduced wound area by about 50% and inflammation (*p* < 0.05), and markedly increased granulation tissue formation by >45%, fibroblast maturation by >28%, and re-epithelialization by >63% compared to control groups (*p* < 0.05), with Nivea producing a superior wound-healing effect. Also, STS supplementation significantly upregulated the expression of CD31 (by >25%), KI-67 (by >25%), CD163 (by >20%), fibronectin (by >14%), and cytokeratin (by >50%) in frostbite wounds compared to control groups, with Aveeno and Nivea producing a better wound-healing effect than Dove and Neutrogena creams. **Conclusions:** In conclusion, STS accelerated healing of frostbite wounds. Therefore, it could be considered as a novel pharmacological treatment of clinical frostbite.

## 1. Introduction

Frostbite injury, also known as freezing cold injury, is a medical condition caused by freezing of the skin and underlying tissues due to extreme cold exposure, occurring at temperatures below 0 °C, with depth and severity being proportional to duration of cold exposure [1,2]. It can affect any part of the body, but the extremities such as fingers and toes, are mostly affected, with frequency up to 90%, and can lead to deep tissue necrosis and amputation, and consequently patient morbidity and disability [2,3,4]. Frostbite injury usually affects military personnel and civilians with predisposing factors such as homelessness, inadequate home heating, alcohol consumption, psychiatric illness, winter sports, drug use, smoking, and smoking-induced vasoconstriction and vascular insufficiency [5,6,7]. According to National Statistics Agency in Canada, where frostbite is commonly experienced in the winter, frostbite injury represented 60.6% of 9425 cold-related hospitalizations from 2011 to 2023 and contributed to increased cold-related hospitalizations by 7.7% during this period [8].

Frostbite injury is classified into four grades based on depth of tissue involvement. Grade 1 is non-full-thickness dermal frostbite, which is characterized by superficial necrotic lesions in the epidermis, mild inflammation, and mild edema in the basal layer of the epidermis and dermis. Grade 2 is full-thickness dermal frostbite, in which necrosis of the epidermal basal layer, Frank edema in the dermis, vascular stasis, and significant inflammation are observed. Grades 3 and 4 represent severe frostbite involving irreversible damage to muscle and bone, and are evidenced by patchy necrosis of cells in the injured area, loss of tissue structure, and major inflammation, which result in severe clinical consequences [9,10]. Recent clinical studies of patients with grades 3 and 4 frostbite injuries revealed a mixed culture of bacteria in frostbite wounds, with Gram-positive (e.g., *Staphylococcus aureus*) and Gram-negative (e.g., *Pseudomonas aeruginosa*) bacteria representing 50.5% and 49.5% of the bacterial population, respectively [11,12]. Frostbite injury is further classified as direct and indirect injury, with the former involving cold-induced injury to skin cells, blood vessels, nerves, muscles, and bones, and the latter involving microcirculatory dysfunction after rewarming [13,14,15]. There are three main mechanisms underlying frostbite injury. They are extracellular and intracellular ice crystallization, intracellular dehydration, and vascular stasis with arterial insufficiency. The formation of extracellular ice crystals damages cell membranes, with consequent cell death. As temperature drops further and freezing continues, water moves out of the cell into the extracellular spaces, resulting in cellular dehydration and intracellular crystal formation and expansion, leading to cell swelling, cell membrane damage, and irreversible ischemic changes. The vascular stasis phase of frostbite injury, also known as the “*adaptive hunting reaction*”, is a paradoxical cyclical vasoconstriction and vasodilation that recur every 5–10 min in the peripheral circulation as a protective mechanism against frostbite injury. It involves vascular shunting, hypoxia, coagulation, thrombus formation, anaerobic metabolism, and tissue death [16,17,18,19].

There is currently no approved pharmacological treatment of frostbite injury besides supportive care and administration of analgesics, leading to suboptimal outcomes. We recently reported that topical application of AP39, a hydrogen sulfide (H_2_S) donor, delivered via a vehicle cream, promotes timely wound healing in a mouse model of acute frostbite injury [20]. Other H_2_S donors, such as JK-1, NaHS, and H_2_S-releasing hydrogel, have also shown beneficial effects in various animal models for cutaneous wound healing similar to frostbite [21,22,23]. Despite the promising results, these H_2_S donors are not approved for clinical use. To facilitate clinical translation, we sought to determine whether sodium thiosulfate (STS), a clinically viable H_2_S donor drug, delivered via commonly used cosmetic creams, also promotes the healing of frostbite wounds in the same mouse model.

## 2. Materials and Methods

### 2.1. Ethical Statement

The animal study protocol was approved by the Animal Use Committee of the University of Western Ontario, Canada (Protocol number 2022-166). The experiment was conducted with consideration of the 3Rs after the protocol was classified as Category D by the Canadian Council of Animal Care’s Categories of Invasiveness for Animal Experiments, adhering to quality assurance according to good laboratory practice and ARRIVE guidelines (Animal Research: Reporting of In Vivo Experiments).

### 2.2. Animal Grouping

Forty C57BL/6 mice aged between 8 and 10 weeks old were purchased from Charles River Canada (Senneville, QC, Canada). The mice were kept under a condition of 12 h light:12 h dark cycle in groups of 5 per cage in the facility of Animal Care and Veterinary Services at an ambient temperature of 21–23 °C and relative humidity of 45–55%. Mice were fed with standard chow diet and tap water ad libitum. Following a 7-day acclimatization period, mice were randomly assigned to 8 groups: Aveeno (n = 5), Dove (n = 5), Neutrogena (n = 5), Nivea (n = 5), Aveeno+STS (n = 5), Dove+STS (n = 5), Neutrogena+STS (n = 5), and Nivea+STS (n = 5).

### 2.3. Skin Preparation

**Figure 1:**
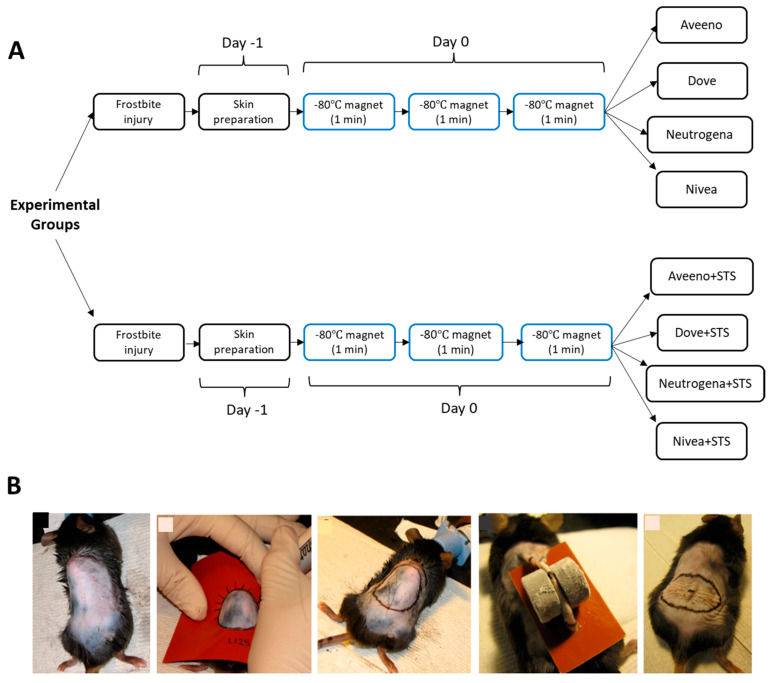
Frostbite injury model showing (**A**) experimental design and (**B**) shaved dorsal skin, circular outline, placement of frozen magnets on dorsal skin, and fresh frostbite wounds immediately after thawing.

In adhering to refinement as part of the 3Rs in animal experimentation, mice were anesthetized by intraperitoneal administration of 100 mg/mL of ketamine and 20 mg/mL of xylazine, and their dorsal fur was shaved with electric clippers. Hair removal cream was applied to remove any remaining fur after shaving. Alcohol prep pads were used to clean the hair removal cream. General anesthesia was maintained by inhaling 5% isoflurane while oxygen was delivered at a rate of 400 mL/minute. While under anesthesia, a silicone sheet and a permanent marker were used to draw a circle on the dorsal skin to serve as a guide to induce frostbite injury, as illustrated in Figure 1.

### 2.4. Preparation of Creams and Their Topical Application

Four readily available and commonly used cosmetic creams (Aveeno, Dove, Neutrogena, and Nivea) were used as vehicle creams to deliver STS, while mice that were treated with these cosmetic creams without STS served as corresponding controls. STS was purchased from the hospital inpatient pharmacy. To prepare the cream+STS, 150 µM STS was added to 1.0 mL of cream using a micropipette and then stirred and vortexed to mix well to ensure stable formulation. The dose of STS was determined from our previous studies to be effective against transplantation-induced ischemia–reperfusion injury (IRI) [24,25,26], which shares pathophysiological similarity with IRI in frostbite injury [27]. According to the manufacturers’ manuals, the cosmetic creams contained the following ingredients, as shown in Table 1 below:

### 2.5. Induction of Frostbite Injury in Mice

Frostbite injury was induced as shown in Figure 1 and previously reported [20,28]. Briefly, two frozen magnets in dry ice (−80 °C) were placed on opposite sides of the skin after a dorsal skin fold was created, as shown in Figure 1B. The magnets were removed after 1 min and replaced immediately with two new frozen magnets followed by another two new frozen magnets to maintain the freezing temperature and a total freezing period of 3 min while core body temperature was taken with a rectal thermometer. The magnets were then removed, and the mice were returned to their room after a short period of recovery in a recovery cage on a heating pad. The frostbite injury was treated with daily topical application of 0.5 mL of Aveeno, Dove, Neutrogena, Nivea, Aveeno+STS, Dove+STS, Neutrogena+STS, and Nivea+STS, starting on day 1, according to the experimental groups. Meloxicam was administered subcutaneously at 1 mg/kg/day for 3 days as post-procedure analgesia. Frostbite images were taken on post-procedure days 4, 7, and 15, and the wound area was measured on days 4 and 15 and quantified using ImageJ software (version 1.52b; National Institutes of Health, USA). As markers of wound healing are best expressed on post-procedure day 15 [20], mice were sacrificed on day 15 after induction of frostbite injury with CO_2_ euthanasia, and the injured tissue samples were harvested and fixed in formalin for histological and immunofluorescence analyses.

### 2.6. Histological Examination

Histological and immunofluorescence analyses were performed as previously described [20]. In brief, 8 μm thick paraffin-embedded skin tissue samples were cut, after which the sections were dewaxed and stained with hematoxylin and eosin. The stained sections were then scored by a pathologist for inflammation, granulation tissue formation, fibroblast maturation, and re-epithelialization based on the following scheme: 0 = None; 1 = Scant; 2 = Moderate; 3 = Abundant [20].

### 2.7. Immunofluorescence Assessment

As previously described [20], immunofluorescence staining was performed using the following antibodies to determine the degree of injury and wound healing: caspase-3 (1:100; apoptotic marker), CD31 (1:100; vascularization and angiogenesis marker), KI-67 (1:50; proliferation marker), fibronectin (1:100; fibroblast marker.), cytokeratin (1:200; keratinocyte marker), and CD163 (1:200; M2-specific anti-inflammatory marker). The antibodies were obtained from Abcam, Toronto, Canada. Skin tissue sections were rehydrated in xylene and a decreasing series of ethanol and de-ionized water. To detect nuclei signals, each tissue section was stained with 300 µL of DAPI (Sigma-Aldrich, Oakville, ON, Canada) followed by a 2 min incubation period at room temperature. Following a washing step with phosphate-buffered saline, the stained sections were mounted with an anti-fade mounting media. Images of the sections from immunofluorescence staining were taken with a fluorescent microscope (Olympus IX83, Concord, ON, Canada) and quantified using ImageJ software (version 1.52; National Institute of Health, USA).

### 2.8. Statistical Analysis

All experimental data are presented as mean ± standard deviation (SD). Statistical analysis was conducted using a t-test and one-way analysis of variance (ANOVA) for multiple comparison, followed by Tukey’s post hoc test with GraphPad Prism software (version 9; La Jolla, CA, USA). *p*-value < 0.05 between groups was considered statistically significant.

## 3. Results

### 3.1. Effect of Supplementation of Aveeno, Dove, Neutrogena, and Nivea with STS in Frostbite Wound Healing

**Figure 2:**
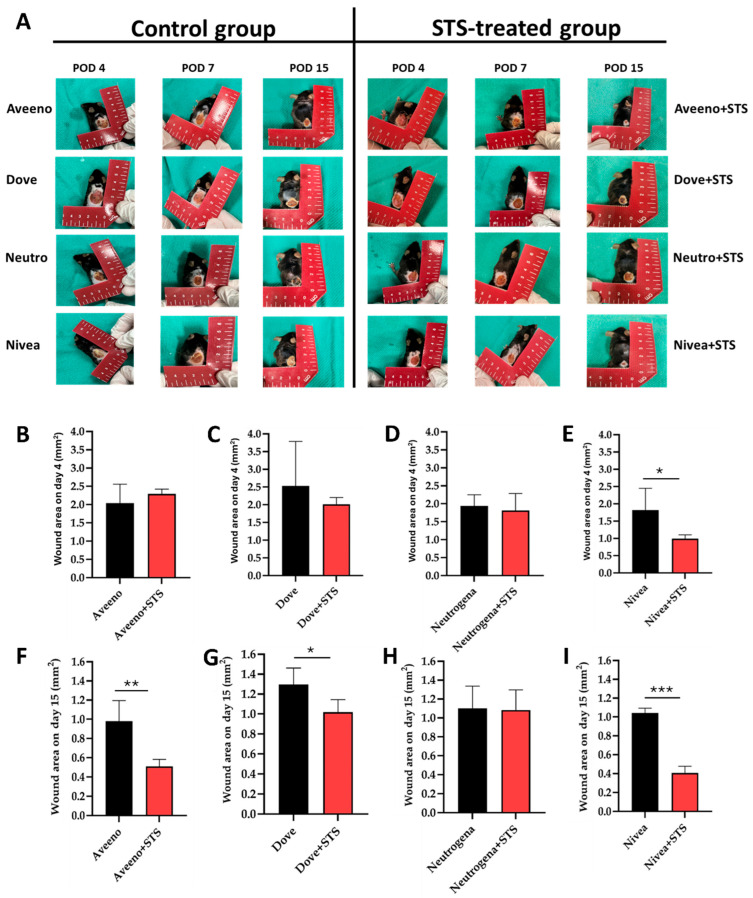
Frostbite wound. (**A**) Progression of frostbite wounds from day 4 to day 15 after induction of frostbite injury. Supplementation of Aveeno, Dove, and Nivea creams with STS enhanced frostbite wound healing and contracture compared to control groups. (**B**–**I**) Quantification of wound area on days 4 and 15 following induction of frostbite injury. * *p* < 0.05 vs. control, ** *p* < 0.01 vs. control, *** *p* < 0.001 vs. control.

**Figure 3:**
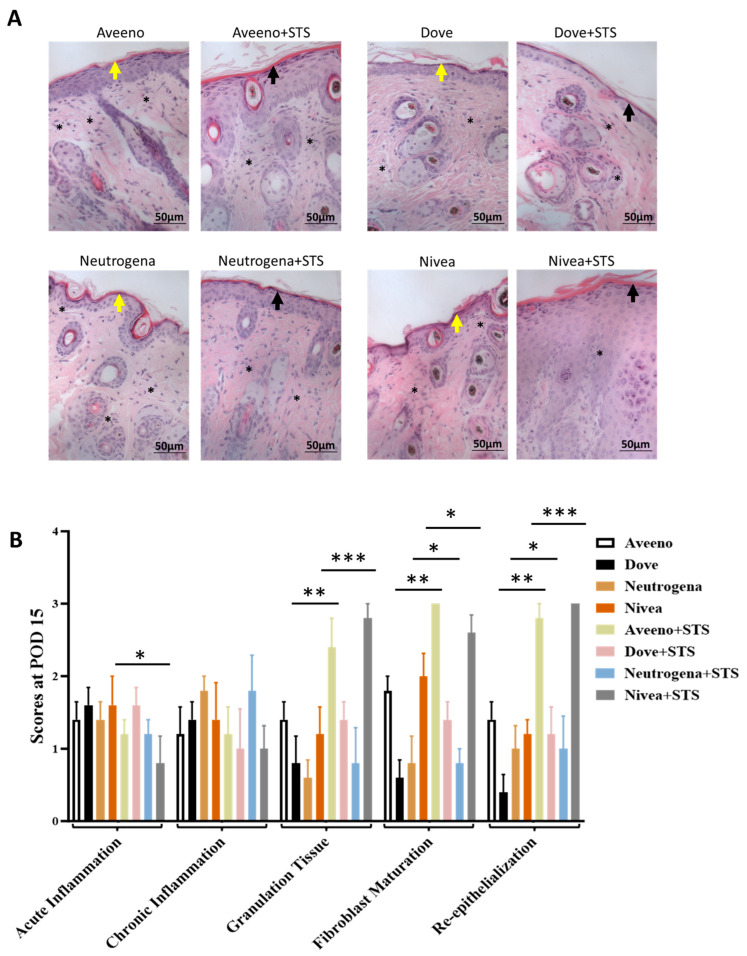
Frostbite-induced regenerative changes in epidermis. (**A**) Histological images at 40× magnification on day 15 after induction of frostbite injury and treatment with Aveeno, Dove, Neutrogena, and Nivea, with STS supplementation. Yellow and black arrows point to regenerative changes, with thin epidermal layer in control group (yellow arrow) and thick layer in STS-treated group (black arrow) and infiltration of dermal inflammatory cells (*). (**B**) Quantification of acute and chronic inflammation, granulation tissue formation, fibroblast maturation, and re-epithelialization. * *p* < 0.05 vs. control, ** *p* < 0.01 vs. control, *** *p* < 0.001 vs. control.

Topical application of Aveeno, Dove, and Nivea supplemented with STS significantly enhanced frostbite wound healing by day 15 after induction of frostbite injury (Figure 2A). This was shown by progressive and marked reduction in wound area relative to control groups without STS supplementation (Figure 2B–I). Interestingly, while addition of STS to Nivea produced the most preferred wound-healing result by day 15 following frostbite injury induction (Figure 2A,E,I; *p* < 0.001), its addition to Neutrogena did not improve frostbite wound healing (Figure 2A,D,H). Also, STS improved regenerative changes in the epidermal layer after the induction of frostbite injury (Figure 3A), characterized by significant improvement in acute inflammation, granulation tissue formation, fibroblast maturation, and re-epithelialization in Nivea vs. Nivea+STS (Figure 3B; *p* < 0.05), Dove vs. Dove+STS (Figure 3B; *p* < 0.05), and Aveeno vs. Aveeno+STS (Figure 3B; *p* < 0.01). In summary, supplementation of Aveeno, Dove, and Nivea creams with STS enhanced the healing of frostbite wounds, with Nivea producing a superior wound-healing effect.

### 3.2. Effect of STS Addition to Aveeno, Dove, Neutrogena, and Nivea on Angiogenesis, Cell Proliferation, Inflammation, and Granulation Tissue Formation in Frostbite Wound Healing

**Figure 4:**
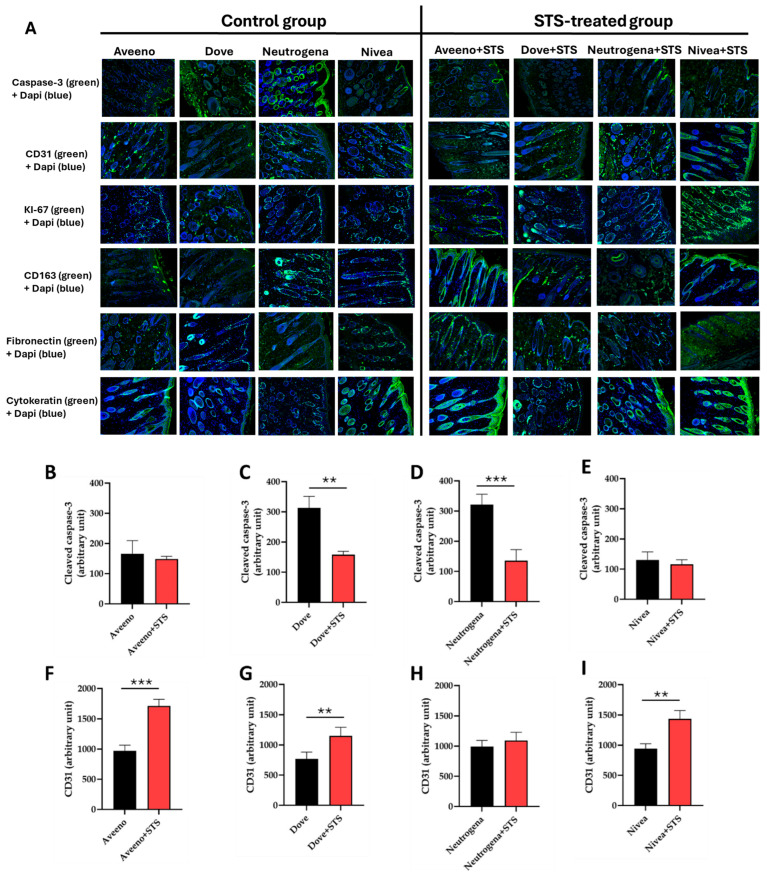

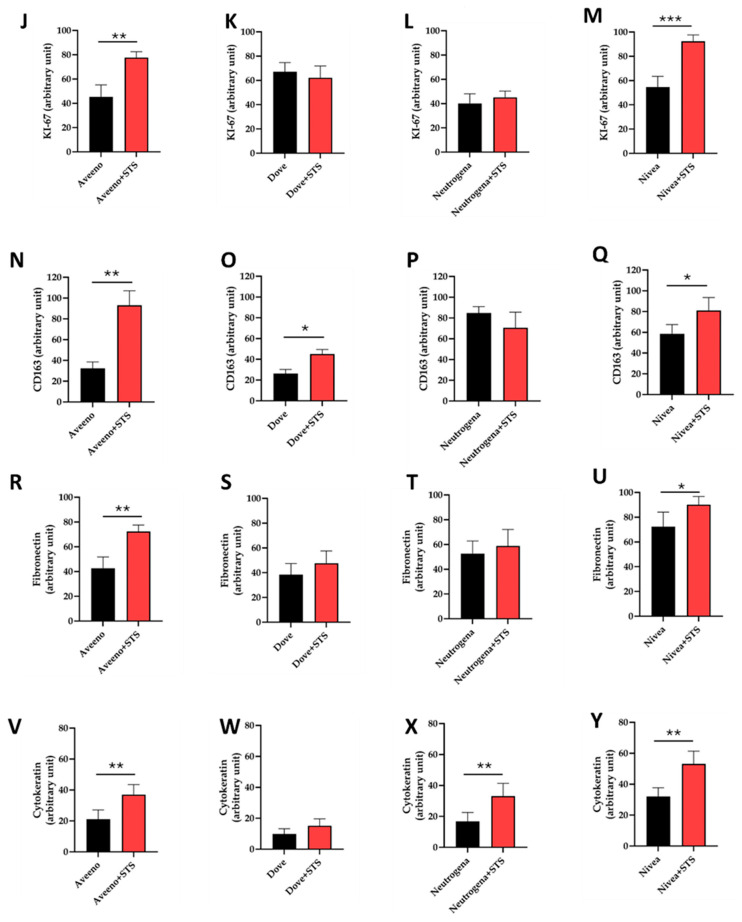
Immunofluorescence and quantification of markers of wound healing showing (**A**) expression of cleaved caspase-3, CD31, KI-67, CD163, fibronectin, and cytokeratin in frostbite wounds treated with Aveeno, Dove, Neutrogena, and Nivea, and their supplementation with STS at 20× magnification. Quantification of immunofluorescence of frostbite wounds treated with Aveeno, Dove, Neutrogena, and Nivea, and their supplementation with STS, indicating the degree of (**B**–**E**) apoptosis, (**F**–**I**) angiogenesis, (**J**–**M**) cell proliferation, (**N**–**Q**) M2-specific anti-inflammation, (**R**–**U**) granulation tissue formation, and (**V**–**Y**) keratinocyte proliferation. * *p* < 0.05 vs. control, ** *p* < 0.01 vs. control, *** *p* < 0.001 vs. control.

Upon induction of frostbite injury, we measured the expression levels of markers of apoptosis (cleaved caspase-3), angiogenesis and vascularization (CD31), cell proliferation (KI-67), M2-specific anti-inflammatory marker (CD163), granulation tissue formation (fibronectin), and keratinocyte differentiation and proliferation (cytokeratin). While cleaved caspase-3 was abundantly expressed in the frostbite wounds of mice that received topical application of Dove and Neutrogena, the expression was downregulated in the wounds of mice that received Aveeno and Nivea treatments (Figure 4A). Interestingly, addition of STS significantly reduced cleaved caspase-3 expression level in the wounds of mice treated with Dove and Neutrogena (Figure 4C,D; *p* < 0.01). However, STS had no effect on cleaved caspase-3 expression in the wounds of Aveeno- and Nivea-treated mice (Figure 4B,E). Also, STS supplementation markedly upregulated the expression of CD31 in frostbite wounds treated with Aveeno, Dove, and Nivea compared to control groups without STS supplementation (Figure 4F–I; *p* < 0.01), whereas no effect was observed in Neutrogena-treated mice (Figure 4H). Remarkably, a similar trend was observed in the expression levels of KI-67, CD163, fibronectin, and cytokeratin (Figure 4J–Y). Collectively, supplementation of Aveeno, Dove, and Nivea with STS improved frostbite wound healing by promoting angiogenesis, cell proliferation and differentiation, anti-inflammation, and granulation tissue formation, with Aveeno and Nivea producing a better wound-healing effect than Dove and Neutrogena creams.

## 4. Discussion

The present study investigated wound-healing performance of STS, an FDA-approved hydrogen sulfide (H_2_S) donor drug, delivered via four commercially available cosmetic creams (Aveeno, Dove, Neutrogena, and Nivea) in a mouse model of acute frostbite injury. As observed in the study, different wound-healing parameters were examined along with histopathological and immunofluorescence assessment on day 15 after induction of frostbite injury, and treatment with or without STS.

Frostbite injury in the present study was associated with infiltration of dermal inflammatory cells, loss of epidermal barrier, damage to the dermal layer, vascular trauma, fluid leakage, and degeneration of panniculus adipose and carnosus. An interesting observation in the present study is the wound-healing effect of both plain creams (control) and STS-loaded creams, with the latter showing significant improvement on day 15 after induction of frostbite injury, except for Neutrogena which appeared to have a lack of effect from STS. While we are unable to explain the lack of effect of STS in the presence of Neutrogena, it is possible that this observation could be due to hydrophilic character, formulation incompatibility, pH, or other reasons which are beyond the scope of our work. As these creams are not medical wound-healing products, there is currently no scientific evidence of their wound-healing effect to support our observation. However, there are reports about certain ingredients in these creams that contribute to cutaneous wound healing. For example, glycerin (a humectant), hyaluronic acid, and provitamin B5 (panthenol; precursor of vitamin B5), in Nivea and Aveeno creams are well-known to create a moist and bacteriostatic environment and enhance regenerative benefits such as stimulation of growth factors and fibroblast and keratinocyte proliferation in the process of cutaneous wound healing [29,30,31,32]. Although our work did not delve into individual components of the creams and detailed examination of synergistic action with STS, it is possible that the observed wound-healing effects of STS-loaded creams and their respective controls (plain creams) could be due to synergism (STS-loaded creams) and individual components (plain creams). Interestingly, supplementation of Nivea cream with STS produced the most preferred wound-healing effect, suggesting that the bioactive ingredients in Nivea (e.g., glycerin, provitamin B5, and hyaluronic acid) might have favored the absorption of STS over the other creams, and significantly activated the cutaneous wound-healing properties of STS compared to the bioactive ingredients in the other cosmetic creams. This is supported by a recent study showing that a hybrid system comprising hyaluronic acid (as contained in our Nivea cream) and JK-1 (an H_2_S donor) contributed to wound repair by upregulating the expression of the M2-like macrophage-specific anti-inflammatory marker [23]. Our observation further suggests that these cosmetic creams could be considered as cosmeceuticals, having bioactive ingredients that show pharmaceutical or medical benefits for the skin, and therefore should be explored further in the fields of cosmeceutical and regenerative medicine.

While supplementation of Aveeno and Dove creams with STS slightly reduced skin inflammation, as shown by our histological data compared to their respective control groups without STS supplementation, the most remarkable decrease in skin inflammation and most significant upregulation of M2-like macrophage-specific anti-inflammatory marker (revealed by our CD163 immunofluorescence staining) was observed in mice that received Nivea cream with STS supplementation. This observation indicates a transition from an inflammatory to a proliferative phase and thereby contributes to accelerated frostbite wound healing, as shown by significantly reduced wound area relative to other groups. This result supports our previous report in which topical application of AP39, a mitochondrially targeted slow-releasing H_2_S donor, delivered through a vehicle cream, promoted wound healing in a similar frostbite model [20]. Considering that STS is also an H_2_S donor, the accelerated wound healing following its addition in the present study is partly via the H_2_S mechanism since the therapeutic benefits of H_2_S are well-established. Hence, it was not surprising that we observed the same salutary effect with STS in the present study as in our previous work.

Our observations further align with previous clinical and preclinical studies which showed that STS exhibits a strong anti-inflammatory action in dermatomyositis and atopic dermatitis by reducing the levels of pro-inflammatory cytokines in the serum of patients as well as immune cell infiltration and inhibition of NLRP3 inflammasome in the skin of mice with atopic dermatitis, which contributed to improved skin condition [33,34,35]. It is important to note that NLRP3 is a well-known key player in inflammatory conditions, contributing significantly to tissue damage, including frostbite. As supplementation of Nivea with STS produced the most effective anti-inflammatory action compared to the other creams in the present study, it suggests that Nivea strongly activated the anti-inflammatory property of STS to promote frostbite wound healing. Although our work did not explore the involvement of cutaneous NLRP3 inflammasome and its downstream targets as the therapeutic mechanism of Nivea with STS supplementation, Karasawa et al. [36] recently reported NLRP3 inflammasome activation and subsequent pro-inflammatory cytokine release in an in vitro model of cryopyrin-associated periodic syndrome, an autoinflammatory condition characterized by cold-induced inflammation similar to frostbite. Therefore, we can surmise that the potent anti-inflammatory action of STS in the presence of Nivea was via NLRP3 inflammasome inhibition.

A crucial part of the complex process of cutaneous wound healing is formation of granulation tissue, which promotes wound healing by secondary intention—filling in the wound from its base to ensure wound contraction and re-epithelialization. Granulation tissue formation is characterized by the presence and proliferation of cells such as fibroblasts, keratinocytes, and endothelial cells [37]. As fibroblasts secrete fibronectin during granulation tissue generation to mediate wound contraction [38], our immunofluorescence data showed significant expression of fibronectin in the frostbite wounds of mice that received topical application of Aveeno and Nivea creams supplemented with STS. Interestingly, topical application of Nivea cream resulted in a higher expression of fibronectin in comparison with the other cosmetic creams in the present study, while its supplementation with STS further upregulated fibronectin expression and contributed to accelerated wound healing by day 15 after induction of frostbite. This implies that STS facilitated cell adhesion, migration, and proliferation in our frostbite model, as downregulation of fibronectin was previously reported to decrease these processes, resulting in delayed wound healing in mouse models of excisional wounds [39] and non-healing radiation wounds [40]. Hence, as further revealed by our immunofluorescence analysis, it was not surprising that in addition to the upregulated fibronectin expression by STS, we also observed consistent corresponding increases in cell proliferation (KI-67 expression) and keratinocyte (cytokeratin) expression upon topical application of Aveeno and Nivea creams supplemented with STS, thus further underscoring the impactful role of STS in rebuilding damaged tissue during the proliferative phase of wound healing. Our observation suggests that STS promotes a balanced and coordinated interaction between fibroblasts, keratinocytes, and wound-healing factors (e.g., growth factors and cytokines), because such an interaction has been shown to facilitate healing in other models of skin wounds [41,42,43,44]. It is important to point out that while supplementation of Aveeno and Nivea creams with STS produced a better wound-healing effect compared to Dove and Neutrogena supplementation in the present study, Aveeno appeared clumpy when supplemented with STS, and therefore, this makes it unsuitable for topical application on human skin. It is possible that STS breaks the chemical bond in Aveeno, hence the clumpy appearance.

Importantly, the increase in cell proliferation and keratinocyte expression observed in the present study is essential for neovascularization and re-epithelialization in the remodeling phase, the final phase of cutaneous wound healing that leads to wound closure and scar formation. As with the markers of wound healing discussed above, neovascularization (CD31 expression) and re-epithelialization were markedly enhanced in Aveeno and Nivea groups that were supplemented with STS. Interestingly, STS also enhanced re-epithelialization in the Dove-treated group. Neovascularization is an important process in the proliferative and remodeling phases of cutaneous wound healing, since the affected frostbite skin loses its blood supply due to severely damaged blood vessels along with increased thrombosis. Thus, the newly formed blood vessels provide nutritive perfusion and oxygen at the wound site, creating and promoting a healthy environment for tissue repair and regeneration. Our observation of enhanced neovascularization and re-epithelialization by STS confirms two recent reports showing that STS stimulated the proliferation of endothelial cells (the building blocks of blood vessels), neovascularization, angiogenesis, and vascular repair following hindlimb ischemia in mice by promoting vascular endothelial growth factor (VEGF; a signaling protein necessary for angiogenesis) [45], and accelerated re-epithelialization and wound closure through its anti-inflammatory and antioxidant activities in a rat model of excision wounds [46].

While we did not investigate the possible molecular mechanisms underlying the wound-healing performance of STS in the present study, our observation can be linked to the H_2_S mechanism, as reported in previous animal models for cutaneous wound healing with various H_2_S donor compounds, including our previous frostbite model [20,21,22,23,47,48]. For example, the hypoxic environment of wounds is known to stimulate angiogenic factors such as VEGF in wound healing [49,50,51], which induces endothelial cell migration in wound healing through vasodilation and promotes endothelial cell proliferation [52]. Interestingly, H_2_S has been identified as a pro-angiogenic factor that stimulates endothelial cell proliferation and migration, upregulates VEGF expression, mediates neovascularization, and interferes with the PI3K/Akt pathway in wound healing as well as in ischemic conditions [48,53,54,55,56]. In addition, results from our previous frostbite model and other animal models for wound healing showed that H_2_S also acts as an anti-inflammatory and pro-proliferative agent which promotes tissue remodeling and wound repair [20,57]. These empirical findings demonstrate that H_2_S-mediated cytoprotective effects in cutaneous wound healing are partly via anti-inflammation, angiogenesis, proliferation, and other therapeutic properties.

Despite the clinical relevance of our work, there are limitations in our study. For example, considering that frostbite victims such as military personnel are exposed to a significantly longer duration of freezing temperatures compared to the duration in our animal model, the immediate shift of the mice to room temperature after induction of frostbite could be a significant contributing factor to their rapid recovery. Unfortunately, we could not evaluate this factor in our mouse model. Also, it is possible that application of the hair removal cream prior to induction of frostbite might have impacted skin permeation and possibly affected penetration of the creams. Therefore, future studies should consider examining the impact of hair removal cream on skin permeation and its interaction with the cosmetic creams. In addition, our study did not include a group of mice with frostbite injury without treatment. This is because our Animal Research Ethics Committee did not approve this group on the grounds of “refinement” since they considered the frostbite injury in our protocol (−80 °C frozen magnets) to cause too much severe pain and distress without treatment. This group would have served as an excellent negative control to separate the effects of the cosmetic creams from the effects of STS.

In conclusion, our work is the first to report the wound-healing performance of STS and these cosmetic creams in experimental frostbite injury. Clinically, while STS is indicated in skin wounds such as those related to calciphylaxis and calcific lesions, its application in frostbite wounds has not been explored. In the present study, we found consistent upregulation of markers of wound healing by STS, accelerating the contracture and healing of frostbite wounds. Hence, the findings from this present study provide experimental support that treatment of frostbite wounds with STS delivered via commercially available cosmetic creams such as Nivea could be a novel and inexpensive approach in pharmacological treatment and management of clinical frostbite wounds. Therefore, as the search for pharmacotherapy for frostbite injury continues, our work provides a preclinical basis for clinical treatment of frostbite wounds with STS and these cosmetic creams. However, this warrants further experimental investigations to understand the mechanisms underlying the wound-healing performance of STS and the cosmetic creams.

## Figures and Tables

**Table 1:** Cosmetic creams and their composition.

Cosmetic Cream	Composition (Active Ingredients)
Aveeno	Glycerin, water, ceramides, cetyl alcohol, shea butter, dimethicone, and hyaluronic acid
Dove	Glycerin, water, dimethicone, stearic acid, mineral oil, niacinamide, and petrolatum
Neutrogena	Glycerin, water, dimethicone, cetearyl alcohol, phenoxyethanol, caprylyl glycol, and ceramides
Nivea	Glycerin, water, mineral oil, lanolin alcohol, microcrystalline wax, hyaluronic acid, panthenol, aluminum stearates, citronellol

## Data Availability

The original contributions presented in the study are included in the article. Further inquiries can be directed to the corresponding author.
